# Probiotic potential of novel *Lactobacillus* and *Limosilactobacillus* isolated from Formosan pangolin feces

**DOI:** 10.1186/s12866-026-04935-7

**Published:** 2026-03-10

**Authors:** Sijia Yu, Peihang Hong, Shyun Chou, Tsui-Fang Liang, Kuei-Shien Lin, Cheng-Hung Lai, Chao-Min Wang

**Affiliations:** 1 Department of Veterinary Medicine, College of Veterinary Medicine, National Chung-Hsing University, 145 Xingda Road, Taichung, 402202 Taiwan; 2 Taiwan Biodiversity Research Institute, 1 Minsheng East Road, Jiji Town, Nantou County, 552005 Taiwan; 3 Department of Veterinary Medicine, College of Veterinary Medicine, National Chiayi University, 580 Xinmin Road, Chiayi, 600023 Taiwan

**Keywords:** Formosan pangolin, *Lactobacillus*, *Limosilactobacillus*, Probiotic potential, Conservation microbiology

## Abstract

**Background:**

The critically endangered Formosan pangolin (*Manis pentadactyla pentadactyla*) faces severe conservation challenges, with gastrointestinal (GI) disorders being a primary driver of mortality in captivity. These ailments are often exacerbated by dietary transitions and anthropogenic stress. While the gut microbiota is crucial for host health, the probiotic potential of the pangolin’s native microbes remains unexplored. This study aimd to isolate lactic acid bacteria (LAB) from wild pangolin feces and preliminarily characterize their probiotic properties.

**Results:**

Ten LAB strains with < 97.3% 16S rRNA gene similarity to known species likely represent undescribed lineages within *Lactobacillus* and *Limosilactobacillus*. Phylogenetic analysis revealed two clusters: three *Lactobacillus* strains related to *L. jensenii* and *L. psittaci*, and seven *Limosilactobacillus* strains close to *L. fermentum*. All isolates showed strong acid and bile tolerance and high cell surface hydrophobicity (> 90%). The *Lactobacillus* cluster exhibited superior auto-aggregation (> 80%), pathogen co-aggregation, and organic acid-mediated antibacterial activity, along with cellular component-driven inhibition of α-glucosidase (66.5–69.4%) and α-amylase (75.8–77.2%). In contrast, *Limosilactobacillus* strains demonstrated metabolite-mediated enzyme inhibition (up to 84.1%) and antioxidant activity (25.6–48.2% TAC; 36.3–46.3% DPPH). All isolates were susceptible to cell wall and protein synthesis inhibitors, confirming a safe antibiotic profile.

**Conclusion:**

These findings identify a reservoir of novel, pangolin-derived LAB with multifaceted probiotic traits. These isolates represent promising candidates for targeted nutritional strategies to mitigate GI distress and improve the survival of this endangered mammal. This work bridges the gap between microbial ecology and practical wildlife conservation, offering a scientific basis for enhancing the health of captive pangolin populations.

**Supplementary Information:**

The online version contains supplementary material available at 10.1186/s12866-026-04935-7.

## Introduction

The Chinese pangolin (*Manis pentadactyla*) is distributed across the northern Indian subcontinent, northern Southeast Asia, and southern China. Three subspecies are recognized: *Manis pentadactyla auritus* in mainland Asia, *Manis pentadactyla pusilla* in Hainan, and *Manis pentadactyla pentadactyla* in Taiwan, commonly referred to as the Formosan pangolin [1]. In 2014, the International Union for Conservation of Nature (IUCN) listed the Chinese pangolin as critically endangered due to intense hunting pressures [2]. Conservation initiatives in Taiwan, launched in 1989, have contributed to a gradual recovery of local populations [3].

Captive breeding is considered an essential strategy for pangolin conservation; however, it remains a formidable challenge. Mortality rates in captivity are alarmingly high, with most individuals dying within six months, and up to 67% within ten days of rescue [4, 5]. Major obstacles include the development of suitable artificial environments and nutritionally adequate diets [6, 7]. Pangolins are obligate insectivores, subsisting on a narrow range of ants, termites, and insect larvae; failure to adapt to artificial diets frequently predisposes them to gastrointestinal disorders [3, 4, 6, 8]. In addition to nutritional constraints, stress arising from suboptimal environmental conditions and human interference further compromise their health, often manifesting as gastric ulcers [6]. The higher cortisol levels observed in adult and captive-born pangolins, compared to sub-adults and rescued individuals respectively, indicat that chronic stress is associated with long-term captivity [9, 10]. Metagenomic analyses further reveal that while short-term captive pangolins maintain a microbiota similar to wild populations, long-term captivity and antibiotic administration drive a marked divergence in microbial composition and a loss of diversity [11]. These findings suggestthat chronic stressors—including inadequate diets, unsuitable environments, and human intervention—may impair gut microbiota via the gut–brain axis, ultimately suppressing immune function and increasing mortality [9].

Members of the genus *Lactobacillus* are abundant in the pangolin gut microbiota, particularly in wild populations [8, 11, 12]. *Lactobacillus* spp. are widely recognized probiotics with well-documented benefits in humans, including mitigation of bacterial vaginosis, atopic dermatitis, and respiratory infections, with their primary application being gastrointestinal health [13–15]. These bacteria enhance intestinal barrier integrity through multiple mechanisms: stimulating mucus secretion, promoting antimicrobial peptide and secretory IgA production, reinforcing tight junctions, and competitively excluding pathogens [16]. They also produce bacteriocins, organic acids, and hydrogen peroxide, which inhibit pathogen colonization and reduce intestinal damage [17]. Additionally, lactobacilli modulate immune responses via cytokine, such as promoting the production of interleukin-10 (IL-10) and regulatory T cell (Treg) to attenuate intestinal inflammation, while enhancing IL-12 secretion to activate natural killer (NK) cells and T helper 1 (Th1) cells for infection defense [16, 18]. Metabolites such as short-chain fatty acids (SCFAs) further suppress pro-inflammatory responses through epithelial receptor signaling [19, 20]. Lactobacilli also exhibit protective effects against antibiotic-associated diarrhea [21] and modulate stress responses by producing neurotransmitters including acetylcholine, nitric oxide, histamine, dopamine, and γ-aminobutyric acid (GABA), thereby alleviating stress-related disorders [22–25]. Restoration of *Lactobacillus* populations has been associated with reduced cortisol levels and improved metabolic and behavioral outcomes in animal models [10, 26].

Given the current conservation challenges facing pangolins and potential benefits of conspecific-origin probiotics, this study aimed to isolate lactic acid bacteria from wild pangolin feces and characterize their probiotic properties. These findings establish a vital foundation for the development of targeted microbial therapies to mitigate GI disorders and enhance the survival of rescued pangolins during rehabilitation and captive management.

## Materials and methods

### *Lactobacillus* species isolation

Eight fecal samples were obtained from eight rescued wild pangolins (Supplementary Table S1): five from the Wildlife Rescue and Research Center, Taiwan Biodiversity Research Institute, and three from Taipei Zoo. Fecal samples were collected using clean trays during the first defecation event after rescue and captivity, then stored in sterile stool specimen containers and frozen. For processing, the central portion of each sample was aseptically scooped with a sterile bamboo stick, homogenized in sterile saline, and then vortexed thoroughly. The mixture was streaked onto de Man, Rogosa, and Sharpe (MRS) agar plates (Difco, France), and incubated anaerobically at 37℃ for 48 h. Colonies were subsequently isolated for pure culture and subjected to Gram staining and microscopic examination. Finally, isolates were stored at -20℃ in MRS broth supplemented with 50% glycerol for further analysis.

### Bacterial identification and phylogenetic analyses

To prepare isolates for DNA extraction, overnight cultures were centrifuged at 12,000 rpm for 5 min to remove residual MRS broth. Genomic DNA was extracted using the phenol-chloroform method with the GenoMaker reagent kit (GenePure Technology, Taichung, Taiwan). The 16 S rRNA gene was amplified by polymerase chain reaction (PCR) using universal primers: forward (5′- AGA GTT TGA TCC TGG CTC AG-3′) and reverse (5′- GGT TAC CTT GTT ACG ACT T-3′) [27]. The PCR products were then sent to Genomics BioSci & Tech. Co., Ltd (New Taipei, Taiwan) for sequencing. The obtained sequences were compared with the GenBank database using the Geneious Prime software (version 2023.2.1). The NCBI GenBank accession numbers of these sequences are PV937035, PV937036, and PV937039–PV937046.

For phylogenetic analyses, 16 S rRNA gene sequences of type strains were retrieved from GenBank. Sequences were aligned using the MAFFT v7.490 plugin [28] in Geneious Prime. The best-fit evolutionary model was determined using ModelFinder [29] implemented in PhyloSuite v1.2.3 [30, 31]. Maximum likelihood (ML) phylogenies were inferred under the GTR + I+G4 + F model, with 1000 ultrafast bootstraps [32] using IQ-TREE v2.2.0 [33].

### Acid and bile tolerance test

Acid and bile tolerance of *Lactobacillus* isolates was evaluated following established protocols [34] with minor modifications. Overnight cultures were centrifuged at 3500 rpm for 10 min and washed three times with sterile phosphate-buffered saline (PBS, pH 7.4). The suspensions (10^7^ CFU/mL) were inoculated into two separate treatments: PBS-buffered MRS broth adjusted to pH 2.5 with hydrochloric acid, and MRS broth supplemented with 0.3% bile (Ox Gall powder, Sigma-Aldrich, Cat. No. B3883). The broths were then incubated at 37 °C. At 0, 1, 2, 4, and 6 h, 100 µL aliquots were taken for ten-fold serial dilution in sterile saline. Triplicate 25 µL aliquots from each dilution were spot-plated onto MRS agar. After 48 h of anaerobic incubation at 37 °C, colonies were enumerated and expressed as log CFU/mL.

### Cell surface hydrophobicity assay

Cell surface hydrophobicity was measured following the method described by Ekmekci et al. [35]. Cell suspensions were prepared as described in the acid tolerance test, and the initial optical density at 600 nm (OD600 nm) was measured as A0 using a microplate reader (Tecan™ Sunrise, Australia). Three milliliters of cell suspension were mixed with 1 mL of toluene (Chung Shing Chemicals Co., LTD., Taiwan) and vortexed for 90 s. The mixture was then incubated at 37℃ for 30 min. After incubation, the aqueous phase was carefully collected, and the OD600 was measured as At in triplicate. PBS served as the blank control for absorbance measurements, recorded as Ap. Hydrophobicity was calculated as:

$$\mathrm{Hydrophobicity} {\%} = (1 - \frac{At - Ap}{A0 - Ap}) \times 100$$

### Auto- and co-aggregation assay

Auto- and co-aggregation assays were performed following the method of Reuben et al. [36]. For auto-aggregation, overnight cultures of *Lactobacillus* species were pelleted, washed with sterile PBS, and adjusted to an OD_600_ of 0.50 ± 0.05 using a cell density meter (Ultraspec 10, Amersham Biosciences). The suspensions were incubated statically at 37℃ for 24 h. At 0, 2, 4, 6, 12, and 24 h, 100 µL of the supernatant was transferred to a 96-well plate in triplicate, and the OD_600_ (At) was measured by microplate reader. The absorbance at 0 h served as the baseline (A0), while PBS functioned as the blank control (Ap). Auto-aggregation was calculated as:

$$\mathrm{Auto-aggregation} {\%} = (1 - \frac{At - Ap}{A0 - Ap}) \times 100$$

For co-aggregation, suspensions of *Escherichia coli* ATCC 25,922, *Pseudomonas aeruginosa* ATCC 27,853 and *Staphylococcus aureus* subsp. *aureus* ATCC 25,923 were prepared similarly. Three milliliters of each *Lactobacillus* species suspension were mixed with 3 mL of pathogen suspension, vortexed, and the initial OD_600_ (A_mix0_) was recorded. After static incubation at 37 °C for 24 h, the supernatant’s OD_600_ (A_mixt_) was measured in triplicate at 2, 4, 6, 12, and 24 h. PBS served as the blank control (Ap). The co-aggregation was calculated as:

$$\text{Co-aggregation } {\%} = (1 - \frac{Amixt - Ap}{Amix0 - Ap}) \times100$$

### Antagonistic activity by agar well diffusion method

The antagonistic activity of *Lactobacillus* species against *E. coli*, *P. aeruginosa*, and *S. aureus* was assessed using the agar well diffusion method [37, 38]. Cell-free culture supernatants (CFCS) were obtained from overnight cultures of *Lactobacillus* species by centrifugation at 3,500 rpm for 10 min, followed by filtration through a sterile Millex^®^-GS syringe filter unit (0.22 μm, Merck KGaA, Germany). Pathogen suspensions (10^8^ CFU/mL) were mixed homogenously with Mueller-Hinton agar (MHA, Difco, France) at a 1:100 dilution before solidification. Wells (7 mm diameter) were created, and 100 µL of either original or neutralized CFCS was added to each. After 20 h of aerobic incubation at 37 °C, the diameter (mm) of inhibition zone was measured. Subsequently, CFCS from strains exhibiting inhibition zones were neutralized to pH 6.5-7.0 using 1mM sodium hydroxide, filter-sterilized, and reassessed via agar well diffusion assay.

### Antibiotic susceptibility test

Antibiotic susceptibility was assessed using the Kirby-Bauer disk diffusion method [39]. Isolate suspensions (10^8^ CFU/mL) were inoculated onto MRS agar using a sterile swab. Antibiotic discs (Liofilchem, Italy, and Oxoid, UK) included amoxicillin (25 µg), bacitracin (10 IU), ceftiofur (30 µg), cephalothin (30 µg), ciprofloxacin (5 µg), clindamycin (2 µg), colistin sulfate (10 µg), doxycycline (30 µg), enrofloxacin (5 µg), florfenicol (30 µg), gentamicin (30 µg), lincomycin (2 µg), trimethoprim-sulfamethoxazole (25 µg), tylosin (30 µg), amikacin (30 µg), amoxicillin-clavulanic acid (30 µg), kanamycin (30 µg), minocycline (30 µg), norfloxacin (10 µg), ofloxacin (5 µg), penicillin G (10 U), polymyxin B (300 IU), and trimethoprim (5 µg). Discs were placed on the agar surface using sterile forceps, and plates were incubated anaerobically at 37 ℃ for 24 h. The diameter (mm) of the inhibition zone was measured.

### Carbohydrate hydrolyzing enzyme inhibitory assay

The inhibitory activities against α-glucosidase (AG) and α-amylase (AA) were evaluated following previously established methodologies [40, 41]. For the α-glucosidase inhibitory assay, 700 µL of CFCS or intact cell suspension (10^8^ CFU/mL) from each strain was mixed with 100 µL of AG enzyme (0.25 U/mL). The mixture was incubated at 37 °C for 15 min, followed by the addition of 100 µL p-nitrophenyl-α-D-glucopyranoside (pNPG, 5 mM) and another 30 min incubation under identical conditions. Then the enzymatic reaction was terminated by adding 1000 µL sodium carbonate (0.1 M). In the α-amylase inhibition assay, 500 µL of CFCS or intact cell suspension (10^8^ CFU/mL) was mixed with 500 µL AA enzyme (1 U/mL), and incubated at 37 °C for 10 min. Subsequently, 500 µL 1% starch (w/v) solution was introduced, and the mixture was further incubated for 10 min. The reaction was halted by adding 1000 µL 3,5-dinitrosalicylic acid (DNS) reagent, followed by a boiling water bath for 5 min. The resulting solution was diluted with 10 mL distilled water prior to spectrophotometric analysis. Negative controls for CFCS and intact cell suspensions were MRS broth and PBS, respectively. Absorbance measurements were performed at 405 nm (AG assay) and 540 nm (AA assay) using a microplate reader. Inhibitory activity (%) was calculated using the formula:

$$\text{inhibitory activity} {(\%)} = [(\mathrm{Ac} - \mathrm{As}) ⁄ \mathrm{Ac}] \times 100$$

where A_S_ and A_c_​ represent the absorbance values of reactions containing the sample and negative control, respectively.

### Antioxidant activity assay

The antioxidant activity of each strain was assessed using both intact cells and CFCS. Overnight cultures were centrifuged (3500 rpm, 10 min), washed and resuspended in PBS to obtain the intact cells, while the supernatants were filter-sterilized to yield CFCS.

Total antioxidant capacity (TAC) and 1-Diphenyl-2-picrylhydrazyl (DPPH) radical scavenging activity were measured using the CheKine™ micro total antioxidant capacity assay kit (Abbkine, USA) and the DPPH free radical scavenging capacity assay kit (SunLong Biotech, China), respectively, according to the manufacturers’ instructions. The intact cell concentration used in the test was 10^6^ CFU/mL. PBS served as the blank control for intact cells, and MRS broth for CFCS. Ascorbic acid was used as positive control. For TAC assay, 10 µL of sample was mixed with 180 µL of working reagent and incubated at room temperature for 5 min, followed by absorbance measurement at 593 nm. For DPPH radical scavenging activity, 10 µL of sample was mixed with 190 µL of working reagent, incubated in the dark at room temperature for 30 min, and absorbance was measured at 515 nm. TAC and DPPH radical scavenging activities were calculated according to the formulas provided in the respective kit instructions.

### Statistical analysis

Prior to performing the two-way analysis of variance (ANOVA), the data were tested to confirm adherence to statistical assumptions. Homoscedasticity was confirmed using Spearman’s rank correlation test, and normality was assessed using standard tests (e.g., Anderson-Darling and Shapiro-Wilk). Given the robustness of ANOVA to minor deviations from normality when variances are equal, parametric tests were maintained. For the two-way ANOVA applied in the time-course assays, the independent variables were explicitly defined as ‘strain type’ and ‘time’. Post hoc multiple comparisons were conducted using Tukey’s test to evaluate the significance of differences between specific groups. Statistical significance was set at *P* < 0.05, and results are presented as mean ± standard deviation (SD).

## Results

### Isolation and Identification of lactic acid bacteria

Through extensive screening of fecal samples from rescued Formosan pangolins (*Manis pentadactyla*), 38 Gram-positive bacterial strains exhibiting typical lactic acid bacteria morphology were isolated. 16 S rRNA gene sequencing revealed ten strains with less than 97.3% sequence similarity to established *Lactobacillus* species in GenBank, suggesting potentially novel lineages. Three strains, including M34 (1427 bp), M43 (1431 bp), and M44 (1425 bp), exhibited sequence similarity to *Lactobacillus jensenii* (95.0–95.7%), *Lactobacillus mulieris* (94.8–95.5%), and *Lactobacillus psittaci* (95.5%). Seven other strains, including M8 (1443 bp), M14 (1421 bp), M30 (1416 bp), M31 (1444 bp), M38 (1415 bp), M41 (1418 bp), and M42 (1389 bp), displayed closest alignments to *Limosilactobacillus fermentum* (95.9–97.3%), *Limosilactobacillus alvi* (96.3–96.6%), and *Limosilactobacillus ingluviei* (95.8–96.1%).

**Fig. 1:**
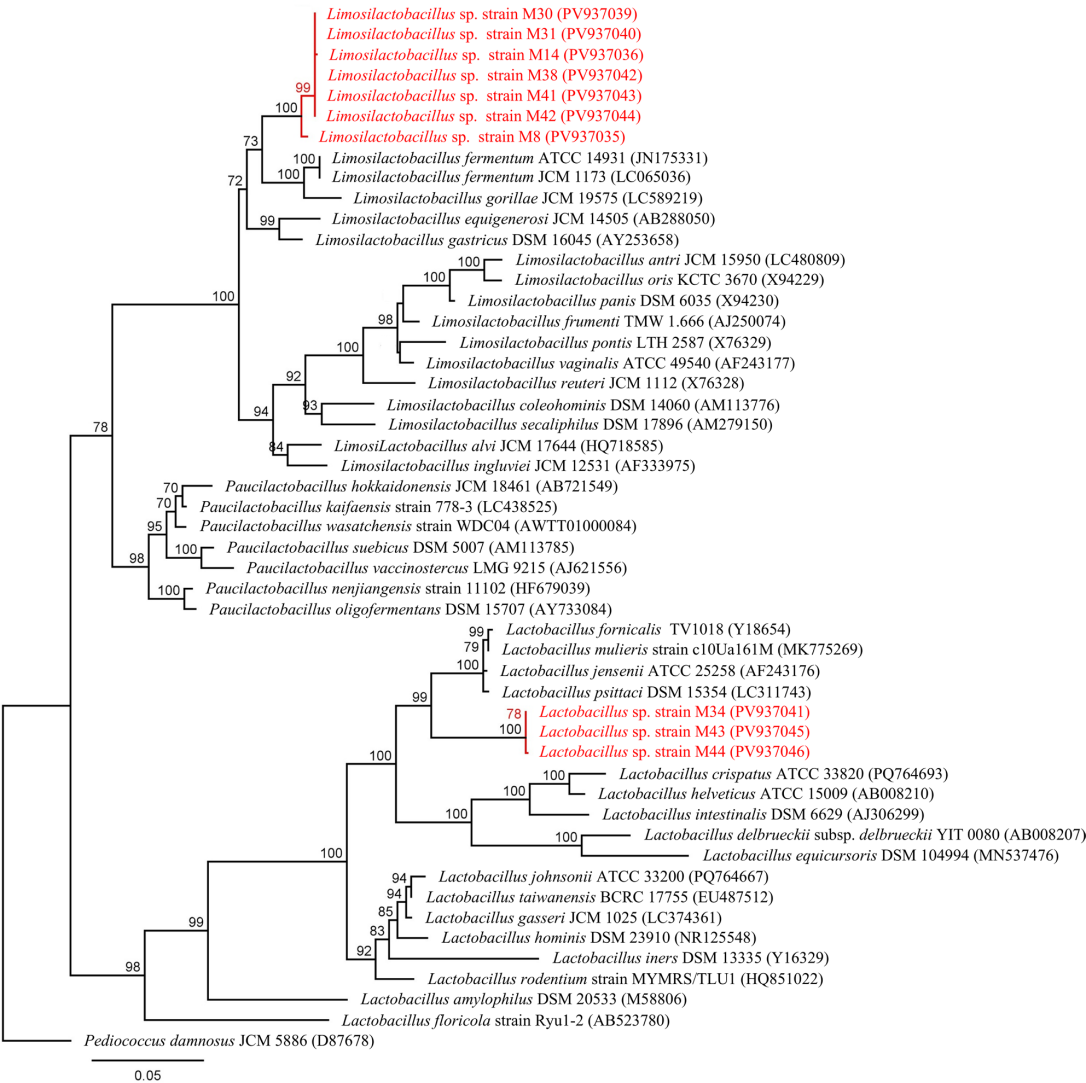
Maximum likelihood (ML) phylogenetic tree based on 16S rRNA gene sequences. Bootstrap values (>70%) calculated for 1000 replicates are shown at branch nodes. Pediococcusdamnosus JCM 5886 was used as an outgroup. Bar, 0.05 substitutions per nucleotide position

A ML phylogenetic tree was constructed using 16 S rRNA gene sequences from type strains of *Limosilactobacillus*, *Paucilactobacillus*, and *Lactobacillus*, with *Pediococcus damnosus* as an outgroup (Fig. 1). The phylogenetic analysis classified these strains into two distinct clades. Strains M34, M43 and M44 formed a sister lineage to a clade containing *L. fornicalis*, *L. mulieris*, *L. jensenii*, and *L. psittaci*, while the remaining seven strains clustered near the clade containing *L. fermentum* and *L. gorilla*. Collectively, these results suggest that the isolates may represent putative novel lineages within the genera *Lactobacillus* and *Limosilactobacillus*, potentially adapted to the unique pangolin gut environment.

### Acid and bile tolerance property

Gastric survival was evaluated by exposing bacteria to pH 2.5 for 6 h. As summarized in Table 1, viable counts of all ten strains decreased under acidic conditions. Strain M34 showed the greatest decline in viability (Δlog CFU/mL = − 2.86), from 10^7.48^ to 10^4.62^ CFU/mL. In contrast, strain M31 demonstrated the highest acid tolerance, with a.

**Table 1:** Acid tolerance property of ten isolates from Formosan pangolin. Viable bacterial counts (Log CFU/mL) in MRS broth at pH 2.5 within 6 h

Isolates	Log CFU/mL				
	0 h	1 h	2 h	4 h	6 h
*Limosilactobacillus* spp.					
M8	7.03 ± 0.02	7.05 ± 0.07	6.84 ± 0.02**	6.76 ± 0.06**	6.70 ± 0.07**
M14	7.31 ± 0.04	7.22 ± 0.01	7.09 ± 0.10**	6.78 ± 0.03**	6.58 ± 0.06**
M30	6.86 ± 0.01	6.73 ± 0.02*	6.64 ± 0.11**	6.50 ± 0.07**	6.40 ± 0.14**
M31	7.49 ± 0.03	7.44 ± 0.00	7.42 ± 0.08	7.38 ± 0.05	7.40 ± 0.07
M38	7.43 ± 0.02	7.33 ± 0.03	7.31 ± 0.03*	7.22 ± 0.02**	7.25 ± 0.03**
M41	7.24 ± 0.04	7.20 ± 0.06	7.10 ± 0.08*	6.42 ± 0.03**	6.01 ± 0.01**
M42	7.40 ± 0.08	7.37 ± 0.04	7.29 ± 0.04	6.92 ± 0.02**	6.37 ± 0.01**
*Lactobacillus* spp.					
M34	7.48 ± 0.04	7.55 ± 0.06	7.52 ± 0.05	6.64 ± 0.03**	4.62 ± 0.07**
M43	7.71 ± 0.05	7.67 ± 0.06	7.54 ± 0.02**	6.74 ± 0.05**	6.65 ± 0.05**
M44	7.68 ± 0.01	7.56 ± 0.06*	7.44 ± 0.07**	7.11 ± 0.06**	7.08 ± 0.11**

Data are presented as mean ± SD (*n* = 3). Statistical significance of differences relative to baseline (0 h) for each strain is indicated: *P < 0.05, **P < 0.01

Δlog CFU/mL value of -0.09, followed by strain M38 (Δlog CFU/mL = -0.18) and M8 (Δlog CFU/mL = -0.33). 

For bile tolerance property, considerable variation was observed among the ten strains (Table 2). Strain M38 increased from 10^7.43^ to 10^8.27^ CFU/mL after six-hour exposure to 0.3% bile. Strains M8 and M41 also showed adaptive growth, with Δlog CFU/mL values of 0.39 and 0.33 respectively. Other strains declined to varying degrees, with strain M14 experiencing the most substantial decrease (Δlog CFU/mL = -1.58). Additionally, strains with relatively good bile tolerance displayed initial reductions followed by regrowth between 4 and 6 h. Overall, the *Lactobacillus* cluster, including strains M34, M43, and M44 exhibited lower tolerance to simulated intestinal conditions than the *Limosilactobacillus* cluster.

**Table 2:** Bile tolerance property of ten isolates from Formosan pangolin. Viable bacterial counts (Log CFU/mL) in MRS broth with 0.3% bile within 6 h

Isolates	Log CFU/mL				
	0 h	1 h	2 h	4 h	6 h
*Limosilactobacillus* spp.					
M8	7.31 ± 0.06	7.31 ± 0.05	7.45 ± 0.07*	7.66 ± 0.06**	7.70 ± 0.03**
M14	7.36 ± 0.05	7.41 ± 0.06	6.83 ± 0.14**	6.48 ± 0.04**	5.78 ± 0.05**
M30	6.89 ± 0.02	6.81 ± 0.02	6.78 ± 0.03	6.56 ± 0.06**	6.68 ± 0.02**
M31	7.46 ± 0.03	7.47 ± 0.03	7.30 ± 0.11**	6.25 ± 0.15**	6.43 ± 0.07**
M38	7.43 ± 0.02	7.36 ± 0.03	7.38 ± 0.05	7.55 ± 0.03*	8.27 ± 0.09**
M41	7.31 ± 0.05	7.24 ± 0.01	7.20 ± 0.08	7.16 ± 0.05*	7.64 ± 0.06**
M42	7.54 ± 0.06	7.49 ± 0.09	7.48 ± 0.04	7.46 ± 0.04	7.46 ± 0.03
*Lactobacillus* spp.					
M34	6.88 ± 0.03	6.70 ± 0.04**	6.66 ± 0.01**	6.55 ± 0.04**	6.59 ± 0.04**
M43	7.64 ± 0.04	7.68 ± 0.01	7.47 ± 0.04**	7.51 ± 0.09*	7.62 ± 0.02
M44	7.61 ± 0.07	7.61 ± 0.04	7.66 ± 0.08	7.53 ± 0.07	7.52 ± 0.06

Data are presented as mean ± SD (*n* = 3). Statistical significance of differences relative to baseline (0 h) for each strain is indicated: *P < 0.05, **P < 0.01

### Cell surface hydrophobicity assay

Toluene-based assays demonstrated hydrophobicity indices exceeding 90% across all ten isolates, indicating strong adhesion potential (Table 3). The *Lactobacillus* cluster exhibited greater cell surface hydrophobicity compared to the *Limosilactobacillus* cluster.

**Table 3:** Cell surface hydrophobicity in toluene

Isolates	Hydrophobicity %
*Limosilactobacillus* spp.	
M8	99.38 ± 0.01
M14	92.23 ± 0.03*
M30	98.04 ± 0.03
M31	96.88 ± 0.02
M38	99.97 ± 0.00
M41	99.92 ± 0.00
M42	100.20 ± 0.01
*Lactobacillus* spp.	
M34	100.88 ± 0.01
M43	100.46 ± 0.00
M44	101.10 ± 0.00

Data are presented as mean ± SD (*n* = 3). *P < 0.0

### Auto- and co-aggregation assay

**Fig. 2:**
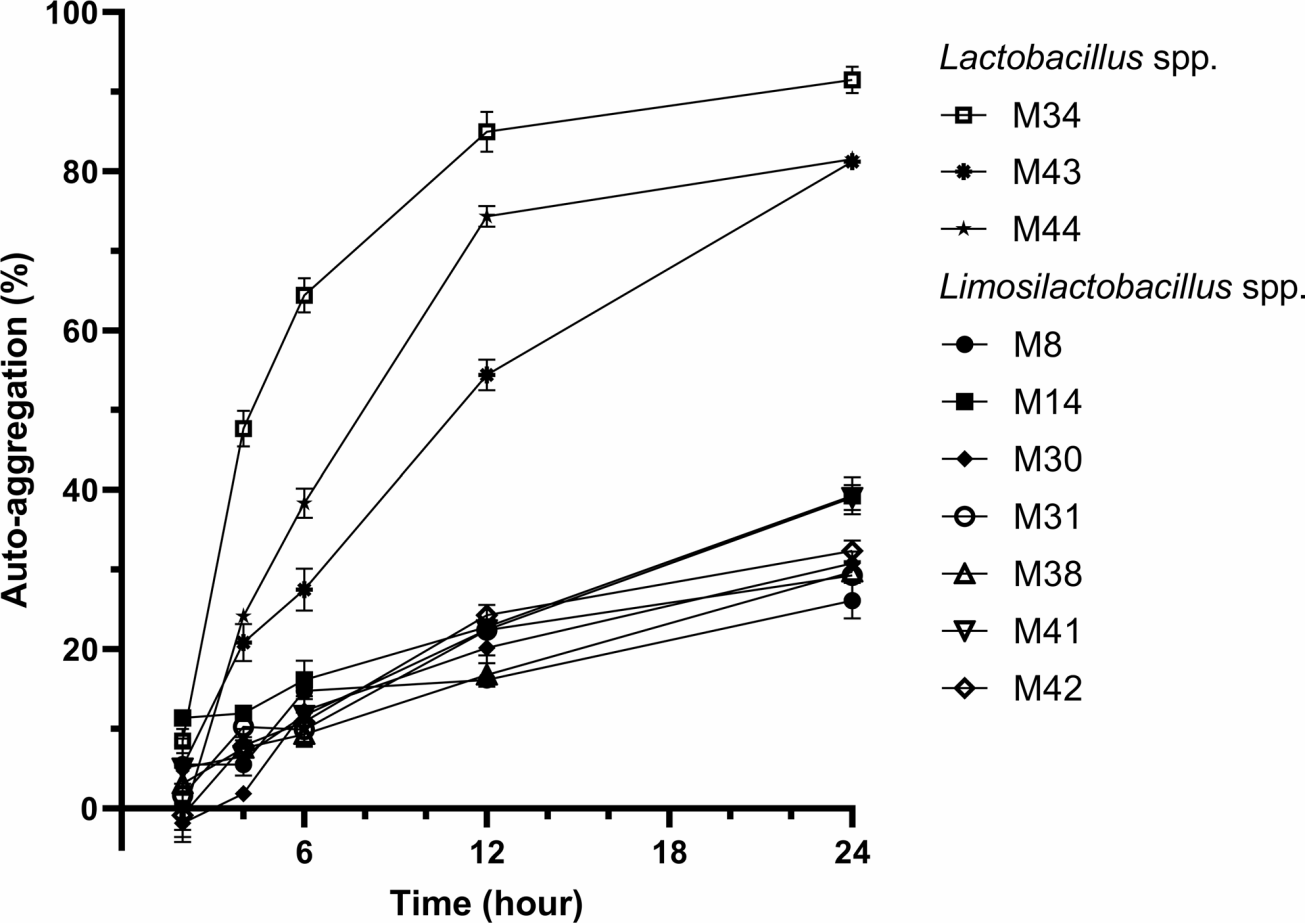
Auto-aggregation assay measured at 2, 4, 6, 12 and 24 hours

Auto-aggregation analysis revealed that the *Lactobacillus* cluster exhibited markedly higher capability than *Limosilactobacillus* strains (Fig. 2). Strain M34 (91.48% ± 1.68) achieved the highest auto-aggregation level after 24-hour incubation, followed by strain M44 (81.50% ± 0.83) and M43 (81.20% ± 0.87), suggesting considerable adhesive potential.

**Fig. 3:**
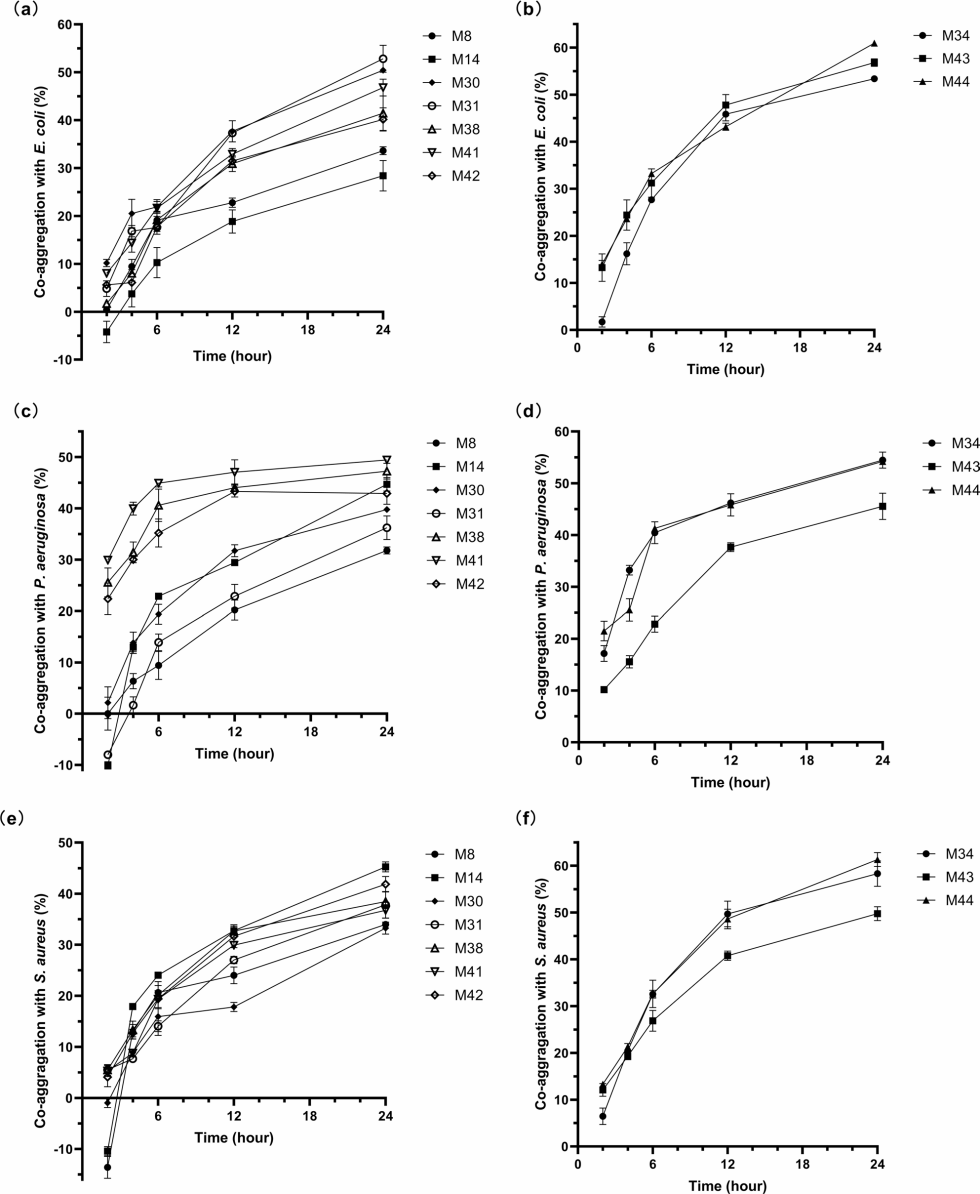
Co-aggregation assay measured at 2, 4, 6, 12 and 24 hours. **a** *Limosilactobacillus *spp. with *Escherichia coli *ATCC 25922; **b*** Lactobacillus *spp. with *E. coli *ATCC 25922; **c** *Limosilactobacillus *spp. with *Pseudomonas aeruginosa* ATCC 27853; **d** *Lactobacillus* spp. with *P. aeruginosa* ATCC 27853; **e** *Limosilactobacillus* spp. with *Staphylococcus aureus* subsp. *aureus *ATCC 25923; **f** *Lactobacillus *spp. with *S. aureus* ATCC 25923

Strain-specific co-aggregation patterns with enteropathogens were also quantified (Fig. 3). *Lactobacillus* strain M44 demonstrated exceptional binding affinity, achieving 60.95% ± 0.50 efficiency against *E. coli* ATCC 25,922, surpassing M43 (56.82% ± 0.79) and M34 (53.40% ± 0.49). In challenges with *P. aeruginosa* ATCC 27,853, strain M34 exhibited the highest co-aggregation (54.45% ± 1.58), slightly exceeding M44 (54.23% ± 0.59). A parallel hierarchy emerged against *S. aureus* ATCC 25,923. M44 maintained dominance (61.33% ± 1.48), while M34 and M43 showed intermediate activity (58.30% ± 2.72 and 49.76% ± 1.48, respectively). Across all pathogens, *Lactobacillus* isolates consistently outperformed *Limosilactobacillus* counterparts, confirming superior pathogen-binding capacity.

### Antagonistic activity by agar well diffusion method

The agar well diffusion method detected strain-specific antimicrobial effects of native acidic CFCS (Table 4). Against *E. coli*, inhibition zones were observed around strain M14 (11 mm), M30 (11 mm), M34 (14 mm), M43 (13 mm), and M44 (15 mm). *P. aeruginosa* inhibition exclusively occurred with M34 and M44, both yielding 12 mm zones. In the presence of *S. aureus*, strain M14, M34, M43, and M44 generated inhibition zones of 13, 15, 12, and 14 mm, respectively. The *Lactobacillus* isolates, particularly M34 and M44, exhibited broad-spectrum inhibition against all three pathogens. Based on these findings, strains M14, M34, M43, and M44 were selected for subsequent evaluation. However, when the CFCS was neutralized, no inhibitory effects were detectable for any strain.

**Table 4:** The inhibition zone diameter (mm) of native acidic CFCS in agar well diffusion test against *E. coli* ATCC 25,922, *P. aeruginosa* ATCC 27,853, and *S. aureus* ATCC 25,923

Isolates	Inhibition zone diameters (mm)^a^			
	E. coli ATCC 25,922	*P*. aeruginosa ATCC 27,853	S. aureus ATCC 25,923	
*Limosilactobacillus* spp.				
M8	-	-	-	
M14	11	-	13	
M30	11	-	-	
M31	-	-	-	
M38	-	-	-	
M41	-	-	-	
M42	-	-	-	
*Lactobacillus* spp.				
M34	14	12	15	
M43	13	-	12	
M44	15	12	14	

^a^ -: absence of a discernible inhibition zone

### Antibiotic susceptibility test

Disk diffusion analysis of 23 clinical antibiotics revealed homogeneous resistance phenotypes among the isolates (Table 5). All strains were uniformly susceptible to cell wall and protein synthesis inhibitors, including penicillins, cephalosporins, tetracyclines, and lincosamides. Conversely, universal resistance was observed against nucleic acid synthesis inhibitors, such as fluoroquinolones, as well as membrane-disrupting agents like polymyxins.

**Table 5:** Antibiotic susceptibility profiles of isolates from Formosan pangolin

Antibiotic	Interpretative zone diameters (mm) ^a^										
	Limosilactobacillus spp.							Lactobacillus spp.			
M8	M14	M30	M31	M38	M41	M42		M34	M43	M44	
Amoxicillin 25 µg	+++	+++	+++	+++	+++	+++	+++		+++	+++	+++
Amoxicillin-Clavulanic acid 30 µg	+++	+++	+++	+++	+++	+++	++		++	++	++
Penicillin G 10 U	+++	+++	+++	+++	+++	+++	+++		+++	+++	+++
Ceftiofur 30 µg	+++	+++	+++	+++	+++	+++	+++		+++	+++	+++
Cephalothin 30 µg	+++	+++	+++	+++	+++	+++	+++		+++	+++	+++
Bacitracin 10 IU	+++	+++	+++	+++	+++	+++	+++		+++	+++	+++
Amikacin 30 µg	N	N	+	+	N	N	N		+	+	N
Gentamicin 30 µg	+	++	+	++	++	+	+		+	++	+
Kanamycin 30 µg	N	N	N	N	N	N	N		N	N	N
Doxycycline 30 µg	+++	+++	+++	+++	+++	+++	+++		+++	+++	+++
Minocycline 30 µg	+++	+++	+++	+++	+++	+++	+++		+++	+++	+++
Tylosin 30 µg	+++	+++	+++	+++	+++	+++	+++		+++	+++	+++
Clindamycin 2 µg	+++	+++	+++	+++	+++	+++	+++		+++	+++	+++
Lincomycin 2 µg	+++	+++	+++	+++	+++	+++	+++		+++	+++	+++
Florfenicol 30 µg	+++	+++	+++	+++	+++	+++	+++		+++	+++	+++
Trimethoprim 5 µg	N	+++	+++	+++	+++	+	N		N	N	N
Trimethoprim-Sulfamethoxazole 25 µg	N	+	+++	+++	+++	N	N		N	N	N
Ciprofloxacin 5 µg	+	N	N	+	N	N	N		N	N	N
Enrofloxacin 5 µg	+	+	+	+	+	+	+		++	+	+
Norfloxacin 10 µg	N	N	N	N	N	N	N		N	N	N
Ofloxacin 5 µg	N	+	+	N	N	N	N		N	N	N
Colistin sulfate 10 µg	N	N	+	N	N	N	N		N	N	N
Polymyxin B 300 IU	N	N	N	N	N	N	N		N	+	N

^a^ 6–8 mm: negative, 9–15 mm: +, 16–19 mm: ++, 20 mm and above: +++

### Carbohydrate hydrolyzing enzyme inhibitory assay

**Fig. 4:**
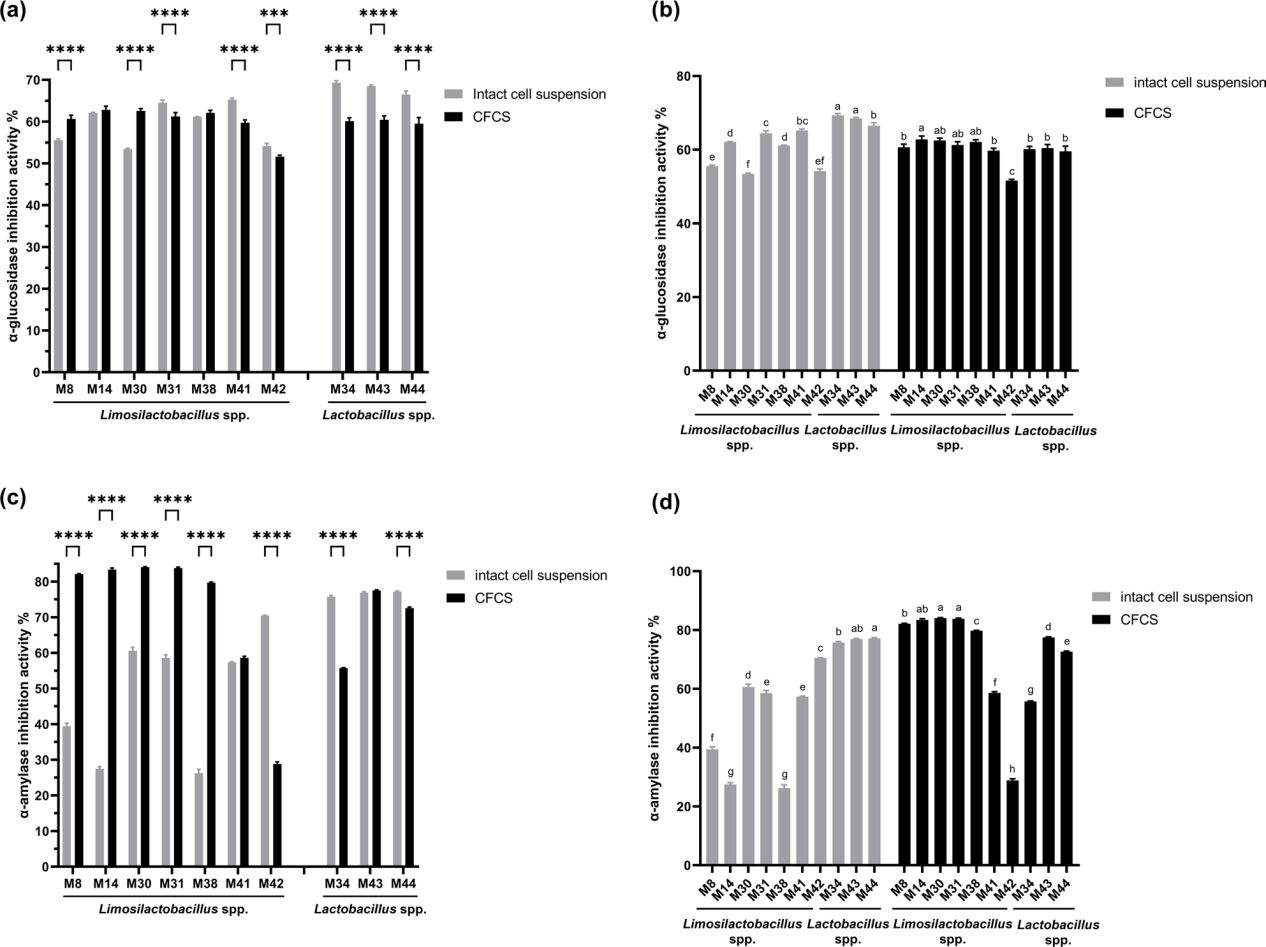
Carbohydrate hydrolyzing enzyme inhibitory assay of intact cell suspensions and CFCS from ten isolates. **a**, **b** α-Glucosidase inhibitory assay and **c**, **d** α-Amylase inhibitory assay. Different letters above the bars indicate statistically significant differences (*p* < 0.05)

All isolates suppressed α-glucosidase activity in both cellular suspensions and CFCS, although efficacy varied significantly among strains (Fig. 4a and b). For intact cells, maximum inhibition occurred with *Lactobacillus* strain M34 (69.37% ± 0.47), followed by M43 (68.50% ± 0.25) and M44 (66.54% ± 0.80), whereas *Limosilactobacillus* M30 showed minimal activity (53.41% ± 0.21). Conversely, CFCS from strain M14 exerted optimal inhibition (62.80% ± 0.87), with M30 (62.56% ± 0.60) and M38 (62.12% ± 0.60) ranking next, while M42 demonstrated lowest efficacy (51.62% ± 0.33). The analysis indicates that the cell suspensions of *Lactobacillus* strains exhibited strong α-glucosidase inhibitory activity, whereas the effect among *Limosilactobacillus* strains was variable.

α-Amylase inhibition displayed distinct preparation-dependent efficacy profiles among strains (Fig. 4c and d). Among cellular suspensions, *Lactobacillus* M44 exhibited dominant activity (77.18% ± 0.23), exceeding M43 (76.92% ± 0.29) and M34 (75.75% ± 0.31), while M38 showed marginal inhibition (26.21% ± 1.10). In contrast, CFCS from M30 generated superior suppression (84.08% ± 0.11), outperforming M31 (83.80% ± 0.27) and M14 (83.41% ± 0.41), whereas M42 was the least effective (28.85% ± 0.58). Collectively, intact *Lactobacillus* cells exhibited stronger α-amylase inhibition, whereas *Limosilactobacillus* strains achieved greater suppression through secreted metabolites.

### Antioxidant activity assay

**Fig. 5:**
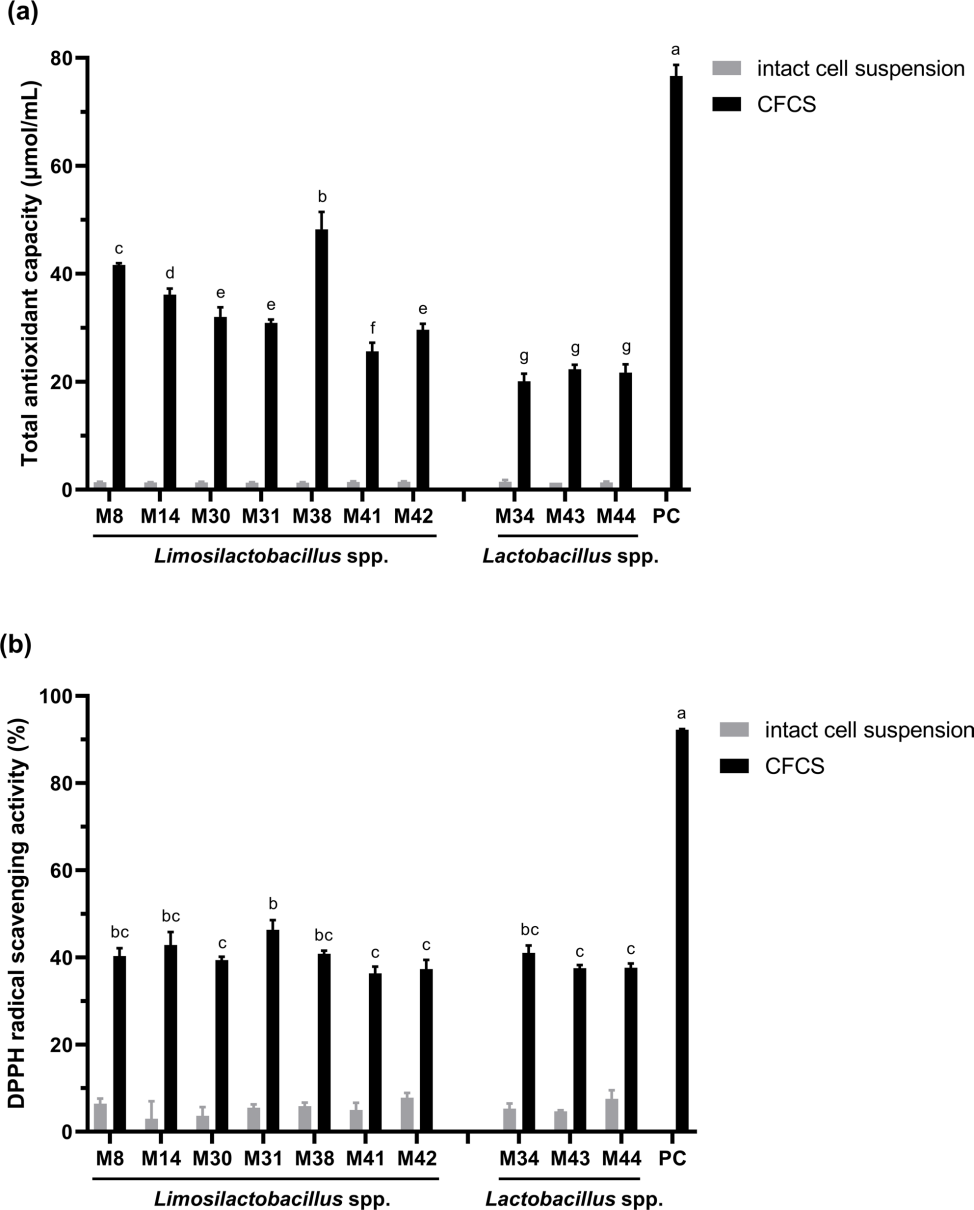
Antioxidant activity assay of intact cell suspensions and CFCS from ten isolates. **a** Total antioxidant capacity and **b** 1-Diphenyl-2-picrylhydrazyl (DPPH) radical scavenging activity. PC, positive control. Different letters above the bars indicate statistically significant differences in the CFCS (*p* < 0.05)

Intact cell suspensions displayed minimal activity in both TAC and DPPH radical scavenging assays (Fig. 5). However, analysis of the CFCS revealed that *Limosilactobacillus* strains consistently surpassed *Lactobacillus* isolates in TAC efficacy. Maximum TAC values were observed for *Limosilactobacillus* M38 (48.23% ± 3.24), followed by M8 (41.61% ± 0.36) and M14 (36.12% ± 1.14), whereas *Lactobacillus* M34 showed minimal performance (20.10% ± 1.38). DPPH scavenging analysis revealed minor differences in the potency of the CFCS among the strains. *Limosilactobacillus* M31 achieved peak antioxidant activity (46.33% ± 2.20), outperforming M14 (42.83% ± 2.98) and M34 (41.01% ± 1.69), with M41 showing minimal efficacy (36.31% ± 1.57). The positive control, ascorbic acid, generated substantially higher values: 76.63% ± 2.08 for TAC and 92.26% ± 0.14 for DPPH scavenging. Overall, CFCS from *Limosilactobacillus* strains demonstrated a slightly superior antioxidant capacity compared to those from *Lactobacillus* strains.

## Discussion

Prior investigations into the gastrointestinal microbiota of *M. pentadactyla* have consistently identified *Lactobacillus* as a predominant genera [10, 11]. To alleviate gastrointestinal disorders in rescued and captive pangolins, this study systematically screened indigenous lactobacilli for probiotic potential. Ten isolates obtained from Formosan pangolin feces demonstrated < 97.3% 16 S rRNA gene sequence identity to established *Lactobacillus* species in GenBank, suggesting they may represent novel species. Based on BLAST results, contemporary *Lactobacillus* classifications, and core genome phylogenies of Lactobacillaceae, a maximum likelihood phylogenetic tree was constructed using 16 S rRNA sequences from these isolates and reference type strains [42]. Phylogenetic analysis revealed two discrete clades. Current evidence indicates that strains M34, M43, and M44 are candidate novel lineages closely related to *L. jensenii* and *L. psittaci*. The remaining seven strains also formed distinct clusters and may represent novel lineages related to *L. fermentum*, although future genomic studies are required for confirmation. Notably, strain M8 exhibited phylogenetic divergence from other *Limosilactobacillus* strains (Fig. 1). This divergence may reflect biogeographical segregation, as strain M8 originated from northern pangolin populations whereas others were derived from central populations. Genomic evidence of Formosan pangolins indicates differentiation between northern and non-northern populations mediated by Xueshan and Central Mountain Range barriers, with northern population exhibiting reduced genetic diversity, elevated inbreeding coefficients, and increased genetic load [43]. Given that host-associated microbiota are shaped by vertical and horizontal microbial transmission within host species, such geographic barriers likely restrict microbial exchange [44]. Consequently, it is plausible that restricted gene flow also drives compositional divergence in pangolin gut microbiomes across geographic regions. Additional variability may stem from differential in captive management protocols among conservation facilities. However, the limited number of bacterial strains included in this study necessitates further investigation via metagenomics to validate the observed geographic clustering. Expanded analyses utilizing high-throughput sequencing data are essential for a systematic characterization of pangolin gut microbial communities across diverse regions.

Comprehensive probiotic screening requires systematic evaluation of gastrointestinal stress tolerance, adhesion ability, antipathogenic activity, safety assessment, and host-associated functional properties [45]. Primary criteria include robust tolerance to digestive stressors such as gastric acidity and biliary secretions. All ten isolates displayed marked acid tolerance relative to the pangolin gastric emptying time (1.34 ± 0.65 h), with strain M44 showing the greatest reduction of 10^0.24^ CFU/mL after 2 h [46]. While strain M34 showed sensitivity after two-hour exposure, others, particularly M31, maintained high stability. Similarly, during simulated small intestinal transit (0.48 ± 0.48 h), all isolates displayed high bile tolerance [46]. Additionally, a reduction of no more than 10^0.28^ CFU/mL in viable cell count was observed in 8 out of 10 strains after 6 h incubation in an Ox Gall-supplemented medium. This suggests that acid and bile tolerance is a conserved trait in these pangolin-derived isolates, ensuring their viability as they reach the distal gut.

Probiotic adhesion, quantified via hydrophobicity and auto–aggregation, mediates primary host–microbe interactions at mucosal interfaces [47]. The strong concordance in these metrics across *Lactobacillus* strain M34, M43, and M44 confirms hydrophobicity as a key determinant of auto–aggregation, highlighting their enhanced adhesive potential [48, 49]. This physical capability likely underpins their superior co-aggregation with pathogens (*E. coli*, *P. aeruginosa*, and *S. aureus*), allowing for competitive exclusion [50, 51]. Furthermore, the CFCS of these strains exhibited broad-spectrum antimicrobial activity, consistent with their co–aggregation capability. The loss of inhibition upon neutralization implicates that organic acids are the primary effectors, which disrupt cellular integrity via proton gradient collapse and osmotic imbalance [52]. However, our current neutralization assays do not rule out the presence of bacteriocins or other proteinaceous antimicrobial compounds. Future studies incorporating specific protease treatments (e.g., trypsin and pepsin) of the cell-free supernatants are required to definitively confirm their involvement.

Safety is paramount for probiotic candidates. Antibiotic susceptibility profiles revealed that all isolates are susceptible to cell wall and protein synthesis inhibitors, while exhibiting intrinsic resistance to aminoglycosides and fluoroquinolones. These resistance patterns align with established *Lactobacillus* profiles [53, 54]. Notably, heterogeneity in diaminopyrimidine susceptibility was observed within the *Limosilactobacillus* cluster, contrasting with the prevalent resistance in lactic acid bacteria from diverse sources [55, 56]. However, similar strain-specific variation are documented in phylogenetically related *L. fermentum* congeners [57]. Although the intrinsic resistance of lactobacilli is well-documented and generally regarded as safe, mandatory genomic screening to eliminate mobile genetic elements remains essential before in vivo probiotic application.

Inhibition of α-amylase and α-glucosidase retards starch and polysaccharide hydrolysis, thereby reducing intestinal glucose absorption rates [58]. In this study, strain-dependent variation was observed between cellular suspensions and metabolite preparations. The *Lactobacillus* cluster demonstrated superior enzymatic inhibition via cellular components, while the *Limosilactobacillus* cluster excelled in metabolite-mediated inhibition, albeit with inter-strain heterogeneity. This aligns with observations in phylogenetically related *L. fermentum* strains, which similarly exhibit metabolite-driven inhibition [59]. Current evidence identifies peptides, glycoproteins, and organic acids as effective constituents conferring inhibitory activity against α-amylase and α-glucosidase [60–62]. While further compositional analyses are required to identify the specific active compounds in our isolates, the potential application is clear. Given that artificial diets typically contain higher carbohydrates levels than wild-type diets, probiotic strategies that inhibit host enzymatic activity offer a promising approach to mitigate hyperglycemia and manage dysbiosis-related metabolic disorders [6, 7].

Antioxidant capacity represents a critical probiotic function. Stress-induced calcium signaling triggers cytochrome c oxidase (COX) dephosphorylation via mitochondrial phosphatases, resulting in excessive production of reactive oxygen species (ROS) [63]. Although the gastrointestinal mucosa is a primary source of ROS, it simultaneously serves as a target for oxidative agents [64]. Excessive ROS impairs intestinal barrier integrity, inducing inflammation associated with various gastrointestinal disorders [64, 65]. Oxidative stress further disrupts gut homeostasis, favoring pro-inflammatory pathogens over beneficial commensals [66]. However, gut bacteria can modulate host oxidative stress through regulated production and activation of antioxidant enzymes [67]. Substantial evidence confirms that lactic acid bacteria generate multiple antioxidant compounds, including superoxide dismutase, glutathione peroxidase, ascorbic acid, melatonin, glutathione, and catalase [45, 66]. These ten isolates from pangolins demonstrated antioxidant activity primarily through metabolite-mediated mechanisms. Thus, probiotic intervention may mitigate oxidative pathology in chronically stressed captive pangolins.

Taken together with the probiotic properties described above, the *Lactobacillus* cluster, phylogenetically close to *L. jensenii*, exhibited high aggregation rates and pathogen inhibition. These findings align with the high adhesive and antimicrobial phenotypes characteristic of *L. jensenii*, which are well-documented mechanisms for maintaining urogenital and vaginal health [68, 69]. In parallel, the *Limosilactobacillus* cluster demonstrated functional consistency with *L. fermentum*, a core species in the Probio-Ichnos database [70]. The ability of these isolates to thrive under acid and bile stress, alongside their antioxidant capacity, mirrors the physiological hallmarks of *L. fermentum* [71, 72]. This benchmarking suggests that these pangolin-derived strains likely share conserved beneficial mechanisms with these well-characterized species.

Despite these promising findings, several limitations of this study warrant consideration. Firstly, the definitive classification of these isolates as novel species is limited by the absence of genomic data, which is essential for a formal taxonomic description. Secondly, the functional characterization remained phenomenological; the specific bioactive molecules conferring antimicrobial, enzymatic inhibition, and antioxidant activities were not purified or structurally identified. Finally, these in vitro assessments, while providing essential foundational evidence, do not fully capture the complexity of the host environment. Future research must evaluate colonization dynamics, persistence, and physiological outcomes in in vivo models to validate the efficacy of these pangolin-specific candidates for practical conservation applications.

## Conclusion

Fecal samples from rescued Formosan pangolins (*Manis pentadactyla pentadactyla*) contained potentially novel lactic acid bacteria lineages with significant in vitro probiotic potential. All isolates exhibited robust viability under simulated gastrointestinal stress and high cell surface hydrophobicity. Distinct functional divergences were observed between phylogenetic clusters. Three *Lactobacillus* strains (M34, M43, M44) demonstrated superior epithelial adhesion, enhanced antipathogenic efficacy, and stronger cellular component-mediated inhibition of carbohydrate-metabolizing enzymes. Conversely, seven *Limosilactobacillus* strains (M8, M14, M30, M31, M38, M41, M42) excelled in metabolite-based enzyme suppression and antioxidant capacity. These strain-specific functional profiles underscore their potential for managing gastrointestinal disorders in captive pangolins. However, translating these in vitro findings into practice requires further validation. Future research must prioritize comprehensive in vivo assessments to establish their efficacy and safety for wildlife conservation, specifically focusing on intestinal colonization dynamics, gut microbiota modulation, and nutrient utilization under artificial dietary regimes.

## Supplementary Information

Supplementary Material 1.

## Data Availability

The datasets generated and/or analysed during the current study are available in the NCBI GenBank repository under accession numbers PV937035, PV937036, and PV937039–PV937046 ( https://www.ncbi.nlm.nih.gov/nucleotide).

## Abbreviations

AA: α-Amylase
AG: α-Glucosidase
CFCS: Cell-free culture supernatants
CFU: Colony forming unit
COX: Cytochrome c oxidase
DNS: 3,5-Dinitrosalicylic acid
DPPH: 1-Diphenyl-2-picrylhydrazyl
GABA: Gamma-aminobutyric acid
IL: Interleukin
IUCN: International Union for Conservation of Nature
LAB: Lactic acid bacteria
MAFFT: Multiple Alignment using Fast Fourier Transform
MHA: Mueller-Hinton agar
ML: Maximum likelihood
MRS: de Man, Rogosa, and Sharpe
NK: Natural killer
PBS: Phosphate-buffered saline
pNPG: p-nitrophenyl-α-D-glucopyranoside
ROS: Reactive oxygen species
SCFA: Short-chain fatty acid
SD: Standard deviation
TAC: Total antioxidant capacity
Th1: T helper 1
Treg: Regulatory T cell
