# Supplementary material for: Probiotic potential of novel Lactobacillus and Limosilactobacillus isolated from Formosan pangolin feces
Source: BMC Microbiol. 2026 Mar 10;26:369. doi: 10.1186/s12866-026-04935-7 (PMC13088532; doi:10.1186/s12866-026-04935-7)
Supplement: Supplementary file 1 — Supplementary Material 1. [file 12866_2026_4935_MOESM1_ESM.docx]

**Table S1** Individual information of sampled pangolins in this study.

| Sample number | Sex | Age class | Location | Fecal quality |
| --- | --- | --- | --- | --- |
| 11207033 | Male | Subadult | Nantou | Normal |
| 11207020 | Female | Subadult | Nantou | Normal |
| 11301031 | Male | Subadult | Nantou | Normal |
| 11306150 | Male | Subadult | Nantou | Normal |
| 11305044 | Male | Adult | Miaoli | Normal |
| 113053101 | Female | Subadult | Taoyuan | Normal |
| 113062501 | Male | Adult | Taoyuan | Normal |
| 113052201 | Male | Subadult | New Taipei | Normal |
